# Advantage of fat-derived CD73 positive cells from multiple human tissues, prospective isolated mesenchymal stromal cells

**DOI:** 10.1038/s41598-020-72012-8

**Published:** 2020-09-15

**Authors:** Eriko G. Suto, Yo Mabuchi, Saki Toyota, Miyu Taguchi, Yuna Naraoka, Natsumi Itakura, Yoh Matsuoka, Yasuhisa Fujii, Naoyuki Miyasaka, Chihiro Akazawa

**Affiliations:** 1 Department of Biochemistry and Biophysics, Graduate School of Health Care Sciences, Tokyo Medical and Dental University, Tokyo, 113-8510 Japan; 2 Department of Urology, Tokyo Medical and Dental University, Tokyo, 113-8510 Japan; 3 Department of Perinatal and Women’s Medicine, Tokyo Medical and Dental University, Tokyo, 113-8510 Japan; 4 Intractable Disease Research Centre, Juntendo University School of Medicine, Hongo 2-1-1, Bunkyo-ku, Tokyo, 113-8431 Japan

**Keywords:** Cell biology, Stem cells

## Abstract

Somatic stem cells have been isolated from multiple human tissues for their potential usefulness in cell therapy. Currently, mesenchymal stromal cells (MSCs) are prepared after several passages requiring a few months of cell culture. In this study, we used a prospective isolation method of somatic stem cells from gestational or fat tissues, which were identified using CD73 antibody. CD73-positive population from various tissues existed individually in flowcytometric pattern, especially subcutaneous fat- and amniotic-derived cells showed the highest enrichment of CD73-positive cells. Moreover, the cell populations isolated with the prospective method showed higher proliferative capacity and stem cell marker expression, compared to the cell populations which isolated through several passages of culturing whole living cells: which we named “conventional method” in this paper. Furthermore, the therapeutic potential of CD73-positive cells was evaluated in vivo using a mouse model of pulmonary fibrosis. After intranasal administration, murine CD73-positive cells reduced macrophage infiltration and inhibited fibrosis development. These results suggest that further testing using CD73-positive cells may be beneficial to help establish the place in regenerative medicine use.

## Introduction

Tissue stem cells reside in various human tissues, including the bone marrow (BM), adipose tissues, placenta, umbilical cord, dental pulp, and synovium^1–3^. Mesenchymal stromal cells (MSCs) from various sources have been widely studied in preclinical trials for potential applications in regenerative medicine and cell therapy. MSCs are characterized by the ability to form fibroblastic colonies, self-renew, and differentiate into several mesenchymal lineages^4^. Owing to their differentiation and immune regulation abilities, MSCs have been tested in approximately 1,000 clinical trials to date for the treatment of multiple conditions, including heart disease, neural diseases, graft-versus-host disease, and autoimmune rheumatic diseases^5–11^. The conventional method to isolate MSCs involves culture with plastic adherence over series of passages, which has been associated with several technical limitations, including high heterogeneity of adherent cells, altered morphological features, and cellular senescence, thereby limiting their clinical potential^12^.

Recent studies have identified cell surface markers that could facilitate MSC isolation, including CD29, CD44, CD90, and CD271^13^. We previously established a method for isolating high-quality BM-MSCs using an anti-CD73 antibody, followed by sorting with flow cytometry; this was termed the “prospective isolation method”^14^. We demonstrated that this method could effectively purify MSCs without requiring long-term culture and passaging. Furthermore, based on selecting with the CD73 antibody, MSCs could be isolated from numerous species, including humans, mice, or rats. CD73 produces adenosine and plays a role in regulating immune tolerance^10,15^. However, collecting cells from the BM is a challenging and invasive procedure, and the BM contains only a small CD73-positive population. Moreover, pooled MSCs obtained from various donors are not suitable for cell transplantation, owing to the higher risk of immune rejection. Therefore, in the present study, we sought to identify a tissue that can be readily accessed with minimal damage to the donor and also contains a high percentage of CD73-positive population.

Toward this end, we isolated CD73-positive cells from various human tissues, including subcutaneous fat, amnion, chorion, villus, and umbilical cord. Using flow cytometry, we evaluated the stem cell characteristics of each population, including the colony-forming ability, proliferation, and expression of MSC markers. To further evaluate the potential of CD73-positive cells as a practical source for cell transplant therapy, we established a mouse model of pulmonary fibrosis using bleomycin (BLM), and examined the impact of intranasal transplantation of CD73-positive cells derived from mouse subcutaneous fat on fibrosis attenuation and on infiltration of inflammatory cells into the lungs.

## Results

### Comparison of CD73-positive cells from tissues of the same patients

**Figure 1:**
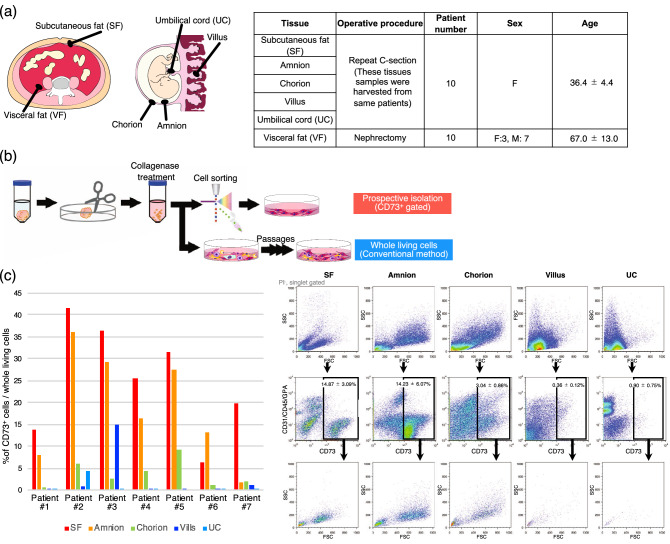
Ratio of CD73-positive cells in human tissues. (**a**) Clinical specimens and their sources obtained from patients, including the subcutaneous fat (SF), amnion, chorion, villus, umbilical cord (UC), and visceral fat (VF). (**b**) Schematic of the experimental procedure for cell isolation. (**c**) Representative profile of flow cytometric analysis for cell surface antigen CD73, CD31, CD45, and glycophorin A (GPA); the bar graph shows the proportion of CD73-positive cells in each tissue.

Subcutaneous fat and placenta were collected from 10 patients who underwent repeat caesarean section, and perirenal fat was collected as visceral fat from 10 patients during nephrectomy (Fig. 1a). The tissues were cut into pieces within 2 h after the operation and treated with 0.2% collagenase for 1 h (Fig. 1b). After filtration, the collected cells were stained with fluorescent-labelled antibodies targeting the mesenchymal cell surface markers CD73, CD29, CD44, CD73, CD90, CD271, CD31, CD45, and CD235a. Dead and doublet cells were gated out, and CD73-positive cells were retained. The percentage of CD73-positive cells from the subcutaneous fat was 14.87 ± 3.09%, which differed from that in visceral fat, 2.04 ± 0.46% (Fig. 1c and Supplementary Fig. S1). Tissues from the placenta (chorion, amnion, umbilical cord, and villus) showed distinct percentages of CD73^+^ cells (Fig. 1c). The proportion appears to be the highest in the amnion and the lowest in the UC. Consistently, CD73-positive cells from the subcutaneous fat and amnion rarely contained hematopoietic cells such as leukocytes or erythrocytes. Interestingly, the size of CD73-positive cells also differed depending on the tissue source (Fig. 1c, Bottom).

### The CD73-positive fraction enriches colony-forming and proliferating cells

**Figure 2:**
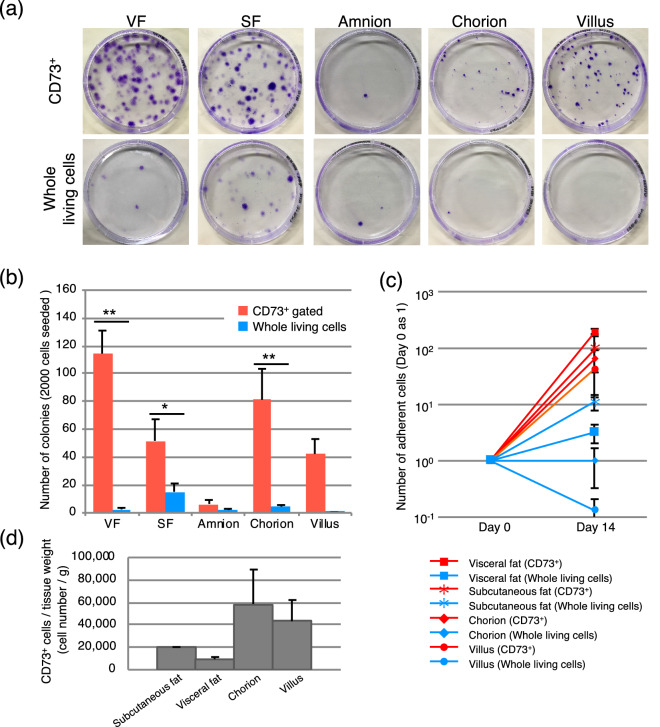
Colony-forming and proliferating abilities of CD73-positive cells. (**a**) Colony-forming assay. Crystal violet-stained colonies of CD73-positive cells and whole living cells from each tissue. A total of 2,000 cells were seeded and cultured for 14 days. (**b**) Number of colonies calculated after culturing 14 days and staining. Error bars are SEM; *p < 0.05, **p 0.01. n = 10, (**c**) Number of proliferating cells over 14 days of culture. The cell number at day 0 was set at 1. n = 4, (**d**) Approximate CD73^+^ cell number from each tissue was counted. n = 4.

A total of 2,000 cells isolated from each tissue were seeded in 10-cm plates and cultured for 14 days, and colony numbers were counted after crystal violet staining. As shown in Fig. 2a,b and supplemental Fig. 2, colony-forming units from the visceral fat, subcutaneous fat, chorion, and villus were enriched in CD73-positive cells, and more MSCs were isolated using the prospective isolation method than that obtained with the conventional method. In line with the results of flow cytometry (Fig. 1c), amnion comprised a large population of CD73-positive cells, although they were predominantly non-adherent cells (Fig. 2a,b and supplemental Fig. 2). However, compared to the cells isolated using the conventional whole-living cell method, more proliferative and adherent cells could be effectively harvested with our prospective isolation approach (Fig. 2c). CD73-positive cells from each wet tissue were counted (Fig. 2d). Depend on tissue amount, it can be calculated estimated cell number after culturing CD73-positive cells.

### Variation in stem cell surface marker expression based on tissue and isolation method

**Figure 3:**
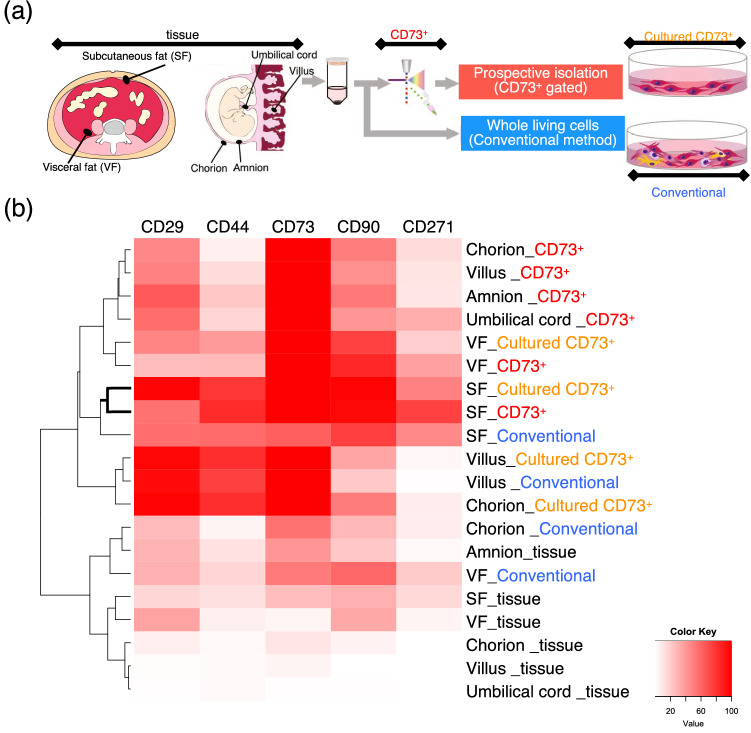
Cell surface marker expression of human tissue-derived cells. (**a**) Cells were harvested at different time points and cell surface marker expression was determined. (**b**) Expression of cell surface markers CD29, CD44, CD73, CD90, and CD271. Four types of cell populations were analysed and compared, whole population immediately after harvesting (tissue), prospective isolated CD73-positive population immediately after sorting (CD73^+^), cultured prospective isolated cells (Cultured CD73^+^), and conventionally harvested cells cultured for 35 days (Conventional). n = 4.

Flow cytometry demonstrated that freshly harvested cells (labelled with the suffix “_tissue” in Fig. 3) rarely expressed MSC markers. However, after prospective isolation (labelled with the suffix “_CD73^+^” in Fig. 3), the same cells highly expressed MSC markers, especially CD44 and CD90. This effect was particularly evident for subcutaneous fat. Moreover, prospective isolated cells from the subcutaneous fat maintained the expression of these markers even after culturing (suffix “_Cultured CD73^+^”). Although cells isolated with the conventional method (suffix “_Conventional”) also expressed MSC markers after culturing, the expression levels were markedly lower than those of the prospective isolated cells.

### Therapeutic potential of CD73-positive cells for pulmonary fibrosis

**Figure 4:**
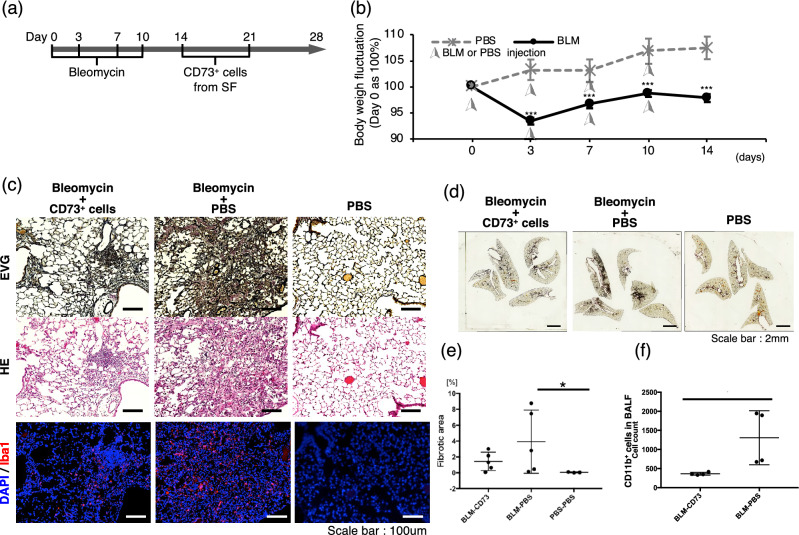
Suppression of fibrosis in mice after transplantation of CD73-positive cells. (**a**) Scheme of CD73-positive cell transplantation. (**b**) Body weight measurement for 14 days (n = 7). (**c**) Representative histological lung sections from bleomycin (BLM)-induced pulmonary fibrosis mice with/without CD73-positive cell transplantation. Phosphate-buffered saline (PBS) was injected as a control. Sections were sliced (5-µm thick) and stained with EVG, hematoxylin and eosin (HE), or immunofluorescent markers (IBA1, red and DAPI, blue). Scale bars, 100 μm. (**d**) Histological analysis of whole lungs. Representative EVG-staining sections are shown. Scale bars, 2 mm. (**e**) Fibrosis area of whole lungs were measured using image J software (version 1.48u4, https://imagej.nih.gov/ij). (**f**) Flow cytometric analysis of infiltrating cells in the bronchoalveolar lavage fluid (BALF) on day 28.

BLM is commonly used for cancer treatment but has a well-known side effect of pulmonary toxicity. Therefore, BLM is widely used for inducing experimental pulmonary fibrosis in mice^16^. In this study, BLM (5 U/kg) was transnasally administered to mice four times (Fig. 4a), and changes in body weight and lung histological sections were analyzed to confirm the development of fibrosis. The BLM group lost significantly more weight compared to the phosphate-buffered saline (PBS)-treated control group (Fig. 4b). Histological analysis further revealed that BLM-treated lungs exhibited alveolar wall thickening, increased collagen deposition, and alveolar wall destruction with air space enlargement (Supplementary Fig. S2, left and middle), confirming successful establishment of the model. Immunofluorescence images showed that IBA1-positive macrophages accumulated in the fibrotic tissue (Supplementary Fig. S2, right).

To verify the therapeutic efficacy of CD73-positive cells, cultured CD73-positive cells from the subcutaneous fat of mice were transnasally inserted twice (on day 14 and day 21 after the first injection of BLM) into the mice. Histological analysis showed that the lung tissues of the CD73-positive cell transplantation model group showed significantly lower Elastic fiber Verhoeff-Van Gieson (EVG) staining than that of the PBS-administered model group (Fig. 4c). There was also a significantly lower number of IBA1-positive macrophages in the CD73-positive cell–transplanted group. Moreover, EVG stained area of lung slices were measured using ImageJ software (https://imagej.nih.gov/ij/) (Fig. 4d,e). CD73-posited cells administered group showed little difference with vehicle control group. To determine the phenotype of infiltrated inflammatory cells in the fibrosis tissue, we sorted the cell types in the bronchoalveolar lavage fluid (BALF) by flow cytometry. The number of CD11b-positive macrophages was reduced in the CD73-positive cells transplantation group (Fig. 4f) compared to the model mice administered PBS as a control. Together, these data suggested that transplantation of CD73-positive cells by transnasal administration could effectively suppress fibrosis by inhibiting inflammation in the lungs of mice.

## Discussion

We previously proposed a prospective isolation method for the effective selection of human BM-MSCs based on CD73 expression^14^. In this study, we further demonstrate that in addition to BM-MSCs, fat or placental MSCs could also be isolated with this prospective isolation method using CD73 antibody. Moreover, we found clear differences in CD73 expression among cells isolated from various tissues, even from the same donors.

Although the precise reason for the difference in CD73-positive cell enrichment in different tissues is currently unclear, the results reflect both individual and tissue effects. To overcome this heterogeneity, we harvested tissues from patients undergoing secondary caesarean sections, and only visceral fat was harvested from patients during perirenal tumorectomy. The visceral and subcutaneous fat showed a greater number of colony-forming cells expressing CD73, indicating a higher CD73-positive cell population in the fat with greater colony-forming ability compared to those of other tissues. In addition to the CD73-positive population ratio, the cell size, cytoplasmic organization, and proliferation rate of these cells showed clear differences among tissues. Even between the fat tissues, MSC markers showed much higher expression in the cells of subcutaneous fat compared with those of the visceral fat. Similarly, there were clear differences in the colony-forming rate and CD73-positive population between the amnion and chorion of the placental tissues. These results suggest tissue-specific characteristics of CD73-positive MSCs, along with notable heterogeneity among individuals for enrichment in the subcutaneous fat and amnion. As clinical studies use MSCs derived from various tissues^10,11,15^, our results highlight the importance of considering the potential different characteristics of the cells depending on the source tissue, which could impact the therapeutic efficacy. CD73-positive cells exhibited CFU-F ability in varying degrees between donors as shown in supplemental Fig. 2. Nonetheless, all CD73-positive population showed high colony forming ability irrespective of ages or tissues. We believe that our prospective isolating method permits stably harvesting of effective cells for transplantation.

In this study, mesenchymal cell marker expression varied among tissues and the isolating method. With respect to the source tissue, the chorion, villus, and umbilical cord of the placenta rarely expressed MSC markers before isolation, whereas the amnion, visceral fat, and subcutaneous fat showed slight expression. After prospective isolation based on CD73 antibody, the placental tissue-derived CD73-postive cells expressed the stem cell marker levels of CD29 and CD90. Prospective isolated visceral fat and subcutaneous fat showed different expression patterns from the other tissues, but were more similar to each other. With respect to the isolation method, the prospective-isolated CD73-positive cells from the subcutaneous fat highly expressed MSC markers such as CD44, CD90, and CD271 compared to conventionally isolated cells. The expression of these markers was also maintained after culturing, and remained at higher levels than conventionally isolated subcutaneous cells. Interestingly, conventionally isolated cells from each tissue located in different clusters based on cell sorting, whereas the prospective isolated cells were all more similar to each other. This indicates that the prospective isolation method could facilitate harvesting homogeneous cells from various tissues.

From a practical perspective, we also found that transplantation of CD73-positive cells isolated from the subcutaneous fat of mice suppressed BLM inhalation-induced lung fibrosis in mice. Fibrosis is the end stage of multiple reactions leading to signaling pathways and dysregulation of inflammatory cells^16^. Pulmonary fibrosis is a progressive lung disease characterized by interstitial fibrosis with a decreasing lung volume^17^. The mechanisms underlying fibrosis progression in cases of idiopathic pulmonary fibrosis (IPF) remain unclear, but appear to involve an excessive immunoreaction. Our results indicate that MSCs have potential to target to the injured area, and to further promote immune tolerance and tissue repair through complex pathways, although the precise mechanism remains to be elucidated. Previous reports have examined the therapeutic effects of human MSC-produced exosomes in a bleomycin-induced model of pulmonary fibrosis and the role of human MSC-secreted soluble factors in a model of emphysesma^18, 19^. In the present our study, it is likely that an additional experimental system of transferring human adipose-derived CD73 cells themselves or their secretions into a mouse model will be necessary. However, due to fundamental species differences and immune rejection, we were unable to transplant the human CD73-positive cells into our model. In vivo experiments using the new experimental model and analysis of exosome differences in different tissues will have important implications for our future experiments.

To obtain highly purified CD73-positive cells is the primary issues for human clinical trials. Currently, widely used methods for antibody-based purification are flow cytometry (FACS) and magnetic activated cell sorting (MACS)^20^. FACS is the preferred method when very high purity of the desired population is required, and when the target cell population expresses a very low level of the identifying marker or when cell populations. Although FACS is frequently used in the laboratory usages, a clinical grade (Good Manufacturing Practice; GMP-grade) flow cytometry has several disadvantages such as low speed, a GMP facility space and cost consuming. MACS has been used in several clinical cases under the guidelines of GMP. CD73 has an advantage that a single antibody can yield cells with high purity. Serial passage of CD73-positive cells over the MACS columns would result in further enrichment. It would be beneficial in clinical application.

Overall, our results showed that CD73-positive cells were effectively harvested from the subcutaneous fat with MSC characteristics. Furthermore, transplantation of these cells suppressed fibrosis and inflammation in pulmonary fibrosis model mice. Although clinical translation of these results requires further detailed research owing to the large gap between a mouse disease model and human disease, our results indicate that CD73-positive cells might provide a new strategy for treating and managing pulmonary fibrosis. Our result indicates that the interstitial pneumonia after COVID-19 infection should be considered for the candidate disease. Further investigations into the detailed mechanisms of pulmonary fibrosis could lead to the development of a safe and effective therapy.

## Materials and methods

### Ethics

All experiments were approved by the local Institutional Review Board of Tokyo Medical and Dental University (No. G2017-005) and all study participants provided written informed consent for use of their materials in research. Visceral fat, subcutaneous fat, amnion, chorion, villus, and umbilical cord tissues were obtained from donors during nephrectomy and caesarean section at Tokyo Medical and Dental University Hospital. All experiments were performed in accordance with relevant guidelines and regulations.

### Cell preparation and culture

After dissection, the tissues were digested with 2 mg/mL collagenase (FUJIFILM Wako Pure Chemical, Osaka, Japan), 10 mM HEPES (Gibco, Carlsbad, CA), 1% penicillin/streptomycin (Gibco), and 7 μg/mL DNase I (Sigma, Saint Louis, USA) prepared in Dulbecco’s modified Eagle medium (DMEM)-GlutaMAX (Gibco) with shaking for 1 h at 37 °C. The digested tissue was filtered through a 70-μm cell strainer (CORNING, Durham NC). Red blood cells were lysed with distilled water and double condensed PBS. After lysing the red blood cells, the remaining cells were suspended in Hanks’ balanced salt solution (HBSS, FUJIFILM Wako Pure Chemical) containing 2% foetal bovine serum (FBS, Hyclone, South Logan, UT), 10 nM HEPES, and 1% penicillin/streptomycin.

### Flow cytometry and cell sorting

The cells diluted in HBSS were stained with the following human antibodies for cell sorting and cell surface analysis: phycoerythrin (PE)-conjugated anti-CD73 (BioLegend, San Diego, CA), allophycocyanin (APC)-conjugated anti-CD29, anti-CD44, APC-conjugated anti-CD73, fluorescein isothiocyanate (FITC)-conjugated anti-CD90, PE-conjugated anti-CD271, PE-Cy7-conjugated anti-CD31, anti-CD45, and anti-CD235a (BD, Franklin Lakes, NJ). Propidium iodide fluorescence was used to gate dead cells. Flow cytometry and sorting were performed on a FACS Aria II instrument (BD).

### Colony-forming unit fibroblast assay

The colony-forming unit fibroblast assay was performed by culturing 2000 sorted cells on a non-coated 100-mm dish for 14 days in DMEM containing 20% FBS (Gibco), 1% penicillin/streptomycin, and 20 ng/mL basic fibroblast growth factor (Repro Cell, Kanagawa, Japan). The medium was changed twice per week. Colonies containing more than 50 cells were counted.

### Mice

Male C57BL/6JJcl mice (9 weeks of age) were purchased from Japan SLC Inc. and C57BL/6-Tg CAG-EGFP mice (8 weeks of age) were purchased from CLEA Japan, Inc. All experimental protocols were approved by the animal committee of Tokyo Medical and Dental University, Japan. All methods were conducted in strict accordance with the approved guidelines of the institutional animal care committee.

### Induction of pulmonary fibrosis

The mice were anaesthetized with 1.5–2% isoflurane (FUJIFILM Wako Pure Chemical) inhalation, and each mouse was set down on its back on an 60°-inclined board with a rubber band running across the upper incisors. BLM-sulfate (5 U/kg body weight; Nippon-Kayaku) in 25 μL PBS was then administered into the left nostril four times to induce lung fibrosis^21^. Control mice were injected with an equal volume of PBS in the same manner.

### Isolation and transplantation of CD73-positive cells

CD73-positive cells were isolated from the subcutaneous fat tissue of 8-week-old C57BL/6-Tg CAG-EGFP mice. Similar to the isolation from human tissues, for collagenase treatments, the subcutaneous fat was chopped and minced in DMEM containing 2% FBS and 2 mg/mL collagenase (FUJIFILM Wako Pure Chemical) for 1 h at 37 °C with shaking. The cells were then diluted in HBSS containing 2% FBS, 10 mM HEPES, and 1% penicillin/streptomycin (Gibco). PI fluorescence was used to remove dead cells, and APC-conjugated anti-CD73 (Biolegend) was used for single-colour staining, followed by sorting with flow cytometry analysis on the FACS Aria II system (BD). The cells were cultured in MSCs medium as previously described^14^. After culture for 14 days, non-adherent cells were removed, and the cells (7.0 × 10^4^ in 25 μL of PBS) were administered to the mice through transnasal injection 14 and 21 days after the first BLM administration. Control animals received an equal volume of PBS.

### Assessment of infiltrating cells in the lung

The mice were sacrificed at days 14 and 28 after BLM administration. The mice were anaesthetised with isoflurane inhalation, and the BALF was collected by instilling 10 mL PBS into the trachea via a 25-gauge needle. The BALF was centrifuged at 800 g for 3 min at 4 °C, and then diluted in HBSS containing 2% FBS, 10 mM HEPES, and 1% penicillin/streptomycin. The BALF was then stained with PE-Cy7-conjugated anti-CD11b (BD) for counting of infiltrating macrophages.

### Hematoxylin and eosin staining

After deparaffinization and rehydration, 5-μm-thick lung tissue sections were stained with hematoxylin solution for 2 min at 50 °C and then rinsed in PBS. The sections were stained with 0.5% eosin solution (FUJIFILM Wako Pure Chemical) for 1 min followed by dehydration with graded alcohol and cleared in xylene.

### EVG staining

After deparaffinization and rehydration, 5-μm-thick lung sections were dipped in 1% acid ethanol (1% HCl in 70% ethanol), stained with Resorcin fuchsin (Muto Pure Chemicals, Tokyo, Japan) for 1 h, and rinsed in PBS. The lung tissues were then stained with hematoxylin solution for 5 min and then with van Gieson solution for 10 min, followed by dehydration with graded alcohol and clearing in xylene.

### Immunofluorescent staining

Five-micrometre-thick sections of the paraffin-embedded lungs were deparaffinized with xylene and hydrated with an ethanol gradient (100–70%). After successively incubating with 1X Target Retrieval Solution (Agilent Dako, Santa Clara, CA) for 30 min, the slides were rinsed with PBS and Blocking One solution for 1 h, and then incubated with the primary antibody against IBA1 (FUJIFILM Wako Pure Chemical) (1:200) overnight at 4 °C. The next day, IBA1 antibody staining was detected with TRITC-labelled goat anti-rabbit IgG (1:200, Thermo Fisher, US). Coverslips were mounted with DAPI (Vector) to identify nuclei.

### Statistical analysis

Data were analysed using the Mann–Whitney U test for variables showing a non-parametric distribution, and with unpaired and two-tailed Student *t*-tests or one-way analysis of variance to compare two or more than two groups, respectively, for variables showing a parametric distribution. All statistical analyses were performed using GraphPad prism 7 software (San Diego, CA, USA). *P* values < 0.05 were considered statistically significant.

## Supplementary information

Supplementary file1

## Data Availability

All data generated or analyzed during this study are included in this published article and its Supplementary Information files.
