# Supplementary material for: Advantage of fat-derived CD73 positive cells from multiple human tissues, prospective isolated mesenchymal stromal cells
Source: Sci Rep. 2020 Sep 15;10:15073. doi: 10.1038/s41598-020-72012-8 (PMC7493914; doi:10.1038/s41598-020-72012-8)

Advantage of fat-derived CD73 positive cells from multiple human tissues, prospective isolated mesenchymal stromal cells

Eriko G. Suto[1,4], Yo Mabuchi[1], Saki Toyota[1], Miyu Taguchi[1], Yuna Naraoka[1,4], Natsumi Itakura[1], Yoh Matsuoka[2], Yasuhisa Fujii[2], Naoyuki Miyasaka[3], and Chihiro Akazawa[1,4*]

[1]*Department of Biochemistry and Biophysics, Graduate School of Health Care Sciences, Tokyo Medical and Dental University, Tokyo 113-8510, Japan*
[2]*Department of Urology, Tokyo Medical and Dental University, Tokyo 113-8510, Japan*
[3]*Department of Perinatal and Women's Medicine, Tokyo Medical and Dental University, Tokyo 113-8510, Japan*
[4]*Intractable Disease Research Centre, Juntendo University School of Medicine, Tokyo 113-8421, Japan*

*Corresponding author
Chihiro Akazawa, MD, Ph.D.
Juntendo University Graduate School of Medicine, Intractable Disease Research Center, Hongo 2-1-1, Bunkyo-ku, Tokyo 113-8431, Japan
Phone number: +81+3-3814-5002, Fax number: +81+3-5802-0439
E-mail address: c.akazawa.gt@juntendo.ac.jp

Supporting information

S1 Figure Legends

Figure S1. The bar graph shows CD73$^+$ cells population in visceral fat from each patient. Representative profile of flow cytometric analysis for cell surface antigen CD73, CD31, CD45, and glycophorin A (GPA).

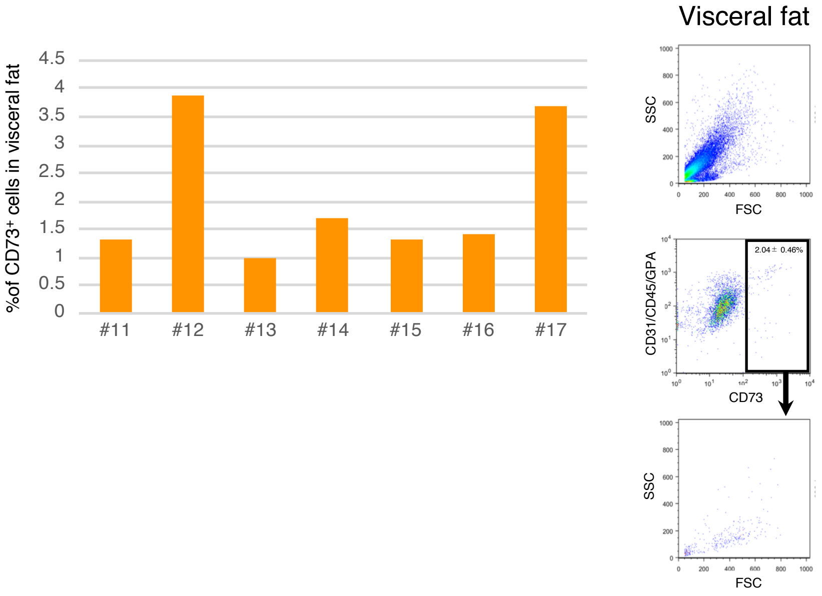

Figure S2. 2000 cells were seeded.(Only cells from umbilical cord #G907 were seeded 765 cells, because CD73+ cells population were rare in umbilical cord.)

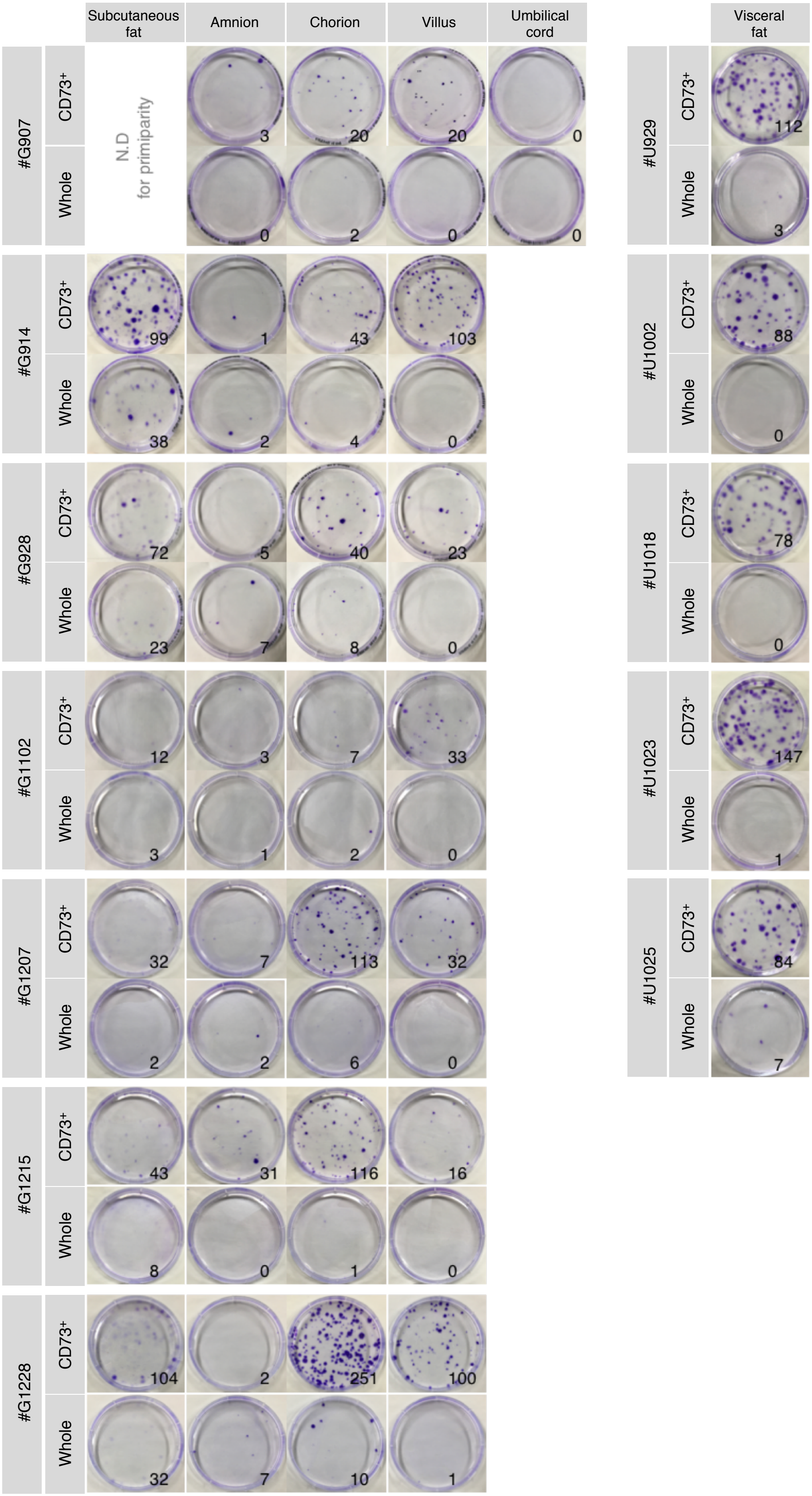

Figure S3. 14days after from first BLM administration, mouse lung was analyzed histologically. Sections were sliced (5-µm thick) and stained with EVG, hematoxylin and eosin (HE), or immunofluorescent markers (IBA1, red and DAPI, blue).

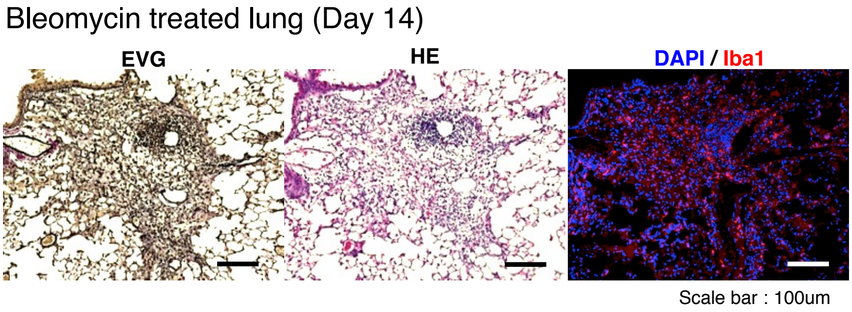

Figure S4. Flow cytometer analysis of infiltrated cells contained in BALF on day 28. Positive cell number of each cell surface marker was counted.

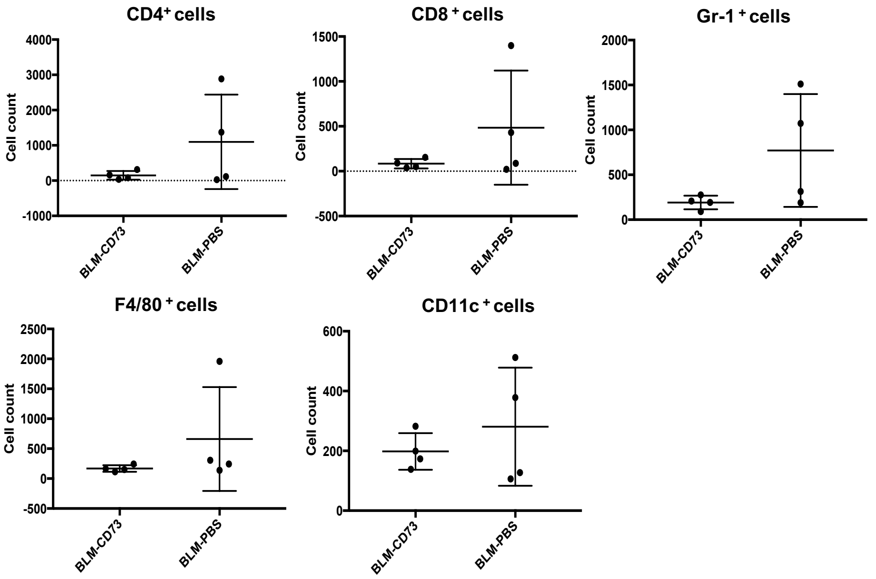

Supplement: Supplementary file 1 — Supplementary file1 [file 41598_2020_72012_MOESM1_ESM.pdf]
